# SARS-CoV-2 Omicron variant in cancer patients: an insight into the vaccine booster debate

**DOI:** 10.2217/fon-2022-0024

**Published:** 2022-02-03

**Authors:** Alessandro Rizzo, Gennaro Palmiotti

**Affiliations:** ^1^Struttura Semplice Dipartimentale di Oncologia Medica per la Presa in Carico Globale del Paziente Oncologico “Don Tonino Bello,” I.R.C.C.S. Istituto Tumori “Giovanni Paolo II,” Viale Orazio Flacco 65, Bari, 70124, Italy

The advent of the SARS-CoV-2 Omicron (B.1.1.529) variant is causing notable concern about the current and future management of the ongoing COVID-19 pandemic, due to the high immune evasion and contagiousness of this variant [1]. The Omicron variant was first isolated in late November of 2021, and it has been suggested that it presents an impressive number of approximately 30–40 mutations in the viral spike protein, resulting in an increased capacity to evade antibodies elicited earlier and to spread more rapidly and extensively than any earlier variant [2]. Based on this evidence, the WHO quickly designated Omicron as a variant of concern [3]. Currently, we are witnessing a dramatic increase in COVID-19 cases due to the Omicron variant, which has resulted in major public health concerns about the enhanced transmissibility of this variant. The Omicron variant has now overtaken the Delta variant in the USA and in several other countries as the predominant strain of the SARS-CoV-2 virus causing COVID-19. Interestingly, this variant does not appear to be causing more severe disease among patients infected with it, although the reasons for that remain unclear [4]; in fact, we do not know yet whether it represents a natural consequence of immunity or Omicron is simply a less virulent pathogen [5]. According to a recent report, the SARS-CoV-2 Omicron variant seems to exhibit resistance to neutralizing antibodies in healthy subjects who have received two doses of mRNA-1273 or BNT162b2 mRNA vaccines [6]. At the same time, healthy recipients of an additional, third mRNA booster vaccine dose seem to exhibit much stronger protection against Omicron, similar to what is observed with other variants of concern. In a recent study, the authors observed that cancer patients represent a key and large group of immunocompromised subjects with lower responsiveness to two-dose mRNA vaccinations [7]; it has been suggested that the use of booster vaccines has the potential to overcome this reduced responsiveness. However, no data are available regarding the impact of booster vaccination against the Omicron variant in immunocompromised patients, including those with hematological and solid tumors. Thus, the breadth of the neutralizing antibody response in these boosted patients – and especially their immunity against the SARS-CoV-2 Omicron variant – is currently unclear [8,9].

Zeng and colleagues recently investigated the neutralizing antibody response to the Omicron variant compared with the Delta and D614G variants among 50 cancer patients in treatment [10], including subjects who had received two mRNA vaccine doses (n = 23) or two doses plus a third booster vaccine (n = 27). The authors showed that following two doses of an mRNA vaccine, the neutralization titer against the Delta and Omicron variants was reduced by fourfold and 21-fold, respectively, compared with D614G. In addition, the report suggested that the Omicron variant may resist neutralization by antibodies elicited by a double dose of either of the mRNA vaccines [10]. At the same time, the authors observed that the neutralizing antibody titers significantly increased following a booster third dose; in particular, increased neutralization for two doses plus a third booster was reported with all variants, and SARS-CoV-2 Omicron variant neutralization was only fivefold less compared with D614G. Moreover, following three doses, no nonresponders against Delta or D614G were highlighted, while approximately a tenth of Omicron assays reported a failure to respond, suggesting that an additional dose could extend the strength and breadth of neutralization, although some patients still do not develop neutralizing antibodies. The authors also investigated whether the use of PD-1 and PD-L1 inhibitors could be a risk factor for low neutralizing response to mRNA vaccines, but no differences were highlighted between two and three vaccine doses, as well as between patients treated with immune checkpoint inhibitors and those who did not receive immunotherapy [10].

In our view, Zeng *et al.* are to be commended for their interesting study on a very timely topic, and positive signals emanating from their report should encourage the scientific community to persist on the road to finding more effective strategies against the COVID-19 pandemic [11,12]. However, several limitations should be considered, including small sample size and a patient population mainly represented by elderly subjects, which often present differences in immune system components compared with younger patients. Further studies are urgently required to confirm the positive impact of a booster dose strategy on protecting cancer patients against the SARS-CoV-2 Omicron variant [13,14]. As we enter year 3 of the ongoing global pandemic, choosing the optimal vaccination strategy to protect the vulnerable population of cancer patients in treatment remains a priority.
